# Autophagy in Femoral Head Necrosis of Broilers Bone Metabolism Parameters and Autophagy-Related Gene Expression in Femoral Head Necrosis Induced by Glucocorticoid in Broilers

**DOI:** 10.3389/fvets.2021.746087

**Published:** 2021-11-02

**Authors:** Kaiyi Pang, Shujie Wang, Meng Li, Zhenlei Zhou

**Affiliations:** Department of Veterinary Clinical Sciences, College of Veterinary Medicine, Nanjing Agricultural University, Nanjing, China

**Keywords:** femoral head necrosis, methylprednisolone, 3-methyladenine, autophagy, broiler

## Abstract

**Objectives:** In this study, the influence of methylprednisolone (MP) and 3-methyladenine (3-MA) on chondrocyte autophagy and bone quality were determined to investigate the mechanisms of femoral head necrosis in broilers.

**Methods:** Chickens were divided into four groups: control, MP, 3-MA, and 3-MA+MP groups. Blood and bone samples were collected for biochemistry assay and bone quality determination. Cartilage was separated from the femoral head for histopathological analysis and gene expression detection.

**Results:** The results indicated that MP treatment significantly affected blood levels of alkaline phosphatase, high-density lipoprotein, calcium, phosphorus, bone alkaline phosphatase, and osteocalcin in broilers. Additionally, MP treatment significantly increased blood levels of cholesterol, low-density lipoprotein, triglyceride, carboxy-terminal telopeptide of type-I collagen, and tartrate-resistant acid phosphatase 5. MP treatment also significantly decreased the levels of bone parameters compared with these values in controls, inhibited the expression of collagen-2, aggrecan, and mammalian target of rapamycin, and increased the expression of beclin1 and microtubule-associated protein 1 light chain 3, hypoxia-inducible factor 1 alpha, phosphoinositide 3-kinase, protein kinase B and autophagy-related gene 5 of the femoral head. Furthermore, following co-treatment with 3-MA and MP, 3-MA mitigated the effects of MP.

**Conclusions:** Our findings demonstrated that autophagy may be involved in the pathogenesis of femoral head necrosis induced by MP in broilers, and this study provides new treatment and prevention ideas for femoral head necrosis caused by glucocorticoids.

## Introduction

The modern poultry industry focuses on selecting fast-growing broilers because of their short feeding period and high feed conversion ratio. However, rapid growth negatively influences the skeletal development of broilers (1). Glucocorticoids (**GCs**) are used extensively to treat chronic inflammatory and autoimmune diseases. However, GC treatment may increase the risk of fractures related to bone fragility and bone loss and cause femoral head necrosis (**FHN**) (2, 3). Therefore, synthetic GCs, such as prednisolone, dexamethasone, and methylprednisolone, have been used to investigate the pathogenesis of FHN in chickens, although the body weight of FHN-affected chickens after GC treatment was lighter than that of birds with naturally occurring FHN (4–12). Our previous study showed that chondrocyte apoptosis in the articular cartilage of the femoral head was an important characteristic of the pathological changes occurring in GC-induced broilers and related genes in endoplasmic reticulum stress (**ERS**) signaling pathway activated to promote apoptosis (13). Autophagy and apoptosis are intricately associated (14). Previous studies reported that different doses of GCs induced autophagy and apoptosis of bone cells (15). In contrast, prolonged autophagy activity precipitates cell apoptosis (16). However, the relationship between GC and autophagy in the femoral head articular cartilage of broilers is unclear.

Autophagy is a leading pathway of programmed cell death under stress conditions. Beclin1 and microtubule-associated protein 1 light chain 3 (**LC3**) genes play crucial roles in autophagy. Beclin1 has been implicated in both the signaling pathway activating autophagy and initial step of autophagosome formation (17). LC3 comprises both a soluble LC3 I and LC3 II (a lipidated form) (18), and LC3 II is considered as one of the most reliable markers of autophagy (19). The hypoxia inducible factor (**HIF**) is transcription factor that mediate the primary transcriptional response to hypoxic stress in normal and transformed cells. HIF-1 has been shown to respond to tissue oxemic state and promote chondrocytes (20). Autophagy-related gene 5 (**ATG 5**) has been characterized as a protein specifically required for autophagy. Protein kinase B (**AKT**) is a serine/threonine protein kinase and is recruited to plasma membrane after activation by phosphoinositide 3-kinase (**PI3K**) and plays important roles in regulating cell growth and apoptosis. A recent study has shown its important role in mediating cell autophagy (21). Inhibition of class III PI3K inhibits autophagy (22).

The autophagy pathway characterized in mammalian species is more systematic than avian species (23). A study revealed that autophagy was overactivated in GC-treated human osteoblasts (24). Autophagy plays an important role in steroid-associated FHN in rats and causes bone loss (25). Articular cartilage is a unique connective tissue that physiologically lacks blood vessels. During the growth of meat-type broilers, mechanical pressure from the body weight on the femoral head rapidly increases, impairing the diffusion of nutrients and oxygen into the articular cartilage (26, 27). This condition affects the autophagy activity of chondrocytes in the cartilage; however, the mechanism is unclear in broilers.

3-methyladenine (**3-MA**) is commonly used as autophagy inhibitor which selectively inhibits class III PI3K to block autophagic activation (28). Study has shown that 3-MA decreased the effect of glucocorticoids induced osteonecrosis of the femoral head in rats (29).

Our previous experiments demonstrated that methylprednisolone (**MP**) can lead to typical FHN in broilers (30). In this study, the functional implications of autophagy in the systemic response were investigated using an MP-induced model, and the autophagic inhibitor 3-MA which inhibited class III PI3K (31) was used to explore the role of autophagy in broilers after GC treatment.

## Materials and Methods

### Animal Treatment and Sample Preparation

All experiments involving animals were performed according to the “Guidelines for Experimental Animals” of the Ministry of Science and Technology (2006, Beijing, China). The procedures were approved by the Institutional Animal Care and Use Committee of Nanjing Agricultural University.

A total of 64 broiler chickens (*Gallus gallus*, AA broilers) of both sexes at 1 day of age were randomly divided into control and experimental groups (MP group, 3-MA group, and 3-MA+MP group) with 16 chickens in each group. The birds were reared under standard conditions and fed a basal diet meeting the National Research Council (1994) requirements (Table 1). From 29 to 35 d of age, the MP group was treated with MP (injection into the pectoralis muscle, 20 mg·kg^−1^, once a day; Enterprise Group Rong Sheng Pharmaceutical Co., Ltd., Henan, China), 3-MA group was treated with 3-MA (injection into the pectoralis muscle, 10 mmol·kg^−1^, once every 2 days; MedChem Express Co., Ltd., Monmouth Junction, MO, USA), and 3-MA+MP group was treated with both 3-MA and MP. The control group was treated with an isodose of sterile saline.

**Table 1:** Ingredients and nutritional levels of elemental diet (air-dry basis) (%).

**Items**	**Content**	
	**Pre-feed (1–21 d)**	**Late feed (22–42 d)**
Ingredients		
Corn	57.00	62.00
Soybean meal	32.60	28.00
Corn gluten meal	3.00	2.00
Soybean oil	3.00	4.00
CaHPO_4_	2.00	1.60
Limestone	1.23	1.30
L-Lys HCl	0.32	0.31
DL-Met	0.15	0.11
NaCl	0.30	0.30
Premix^*^	0.40	0.38

* *Premixes are provided for per kilogram diet as follows: vitamin A (12,000 IU), vitamin D3 (3,000 IU), vitamin E (30 IU), vitamin K3 (1.3 mg), vitamin B12 (0.013 mg), thiamine (2.2 mg), riboflavin (8 mg), niacin (40 mg), choline chloride (400 mg), calcium pantothenate (10 mg), pyridoxine (4 mg), biotin (0.04 mg), folic acid (1 mg), iron (80 mg), copper (7.5 mg), manganese (110 mg), zinc (65 mg), iodine (1.1 mg), selenium (0.3 mg)*.

At 42 d of age, the ability of bird to walk was scored on a six-point scale (32). All birds were weighed, and blood samples were collected from the jugular vein. Then, birds were killed by cervical dislocation. Bone samples, including the femur, tibia, and humerus, were collected and cleaned of all adherent tissue. Since the two legs of the same chicken may have different symptoms, both legs of each chicken were evaluated for FHN (33).

The femoral head was cut along the sagittal plane. One half was rinsed with PBS and fixed in 4% paraformaldehyde at 4°C, and the other half was treated with 0.1% diethyl pyrocarbonate (DEPC, a nuclease inhibitor, particularly against ribonucleases) and then preserved at −80°C until analysis.

### Radiographic Density and Mechanical Tests

Bone mineral density (**BMD**) was measured with an InAlyzer (Medikors, Inc., Gyeonggi-do, Korea), the average density of the entire bone was used. The femur, tibia and humerus of all 64 chickens were tested. Before measuring bone strength, the weight, length, and diameter of the bones were measured. The bones were loaded to failure in three-point bending fracture tests (LR10K PLUS, Lloyd Instruments, Ltd., Hampshire, UK) (34). Each bone sample was positioned on the middle portion of diaphysis, which is the point with maximum stability. A vertical load of 15 mm/min was applied and remained constant until failure. The values were registered and analyzed using software (NEXYGEN Plus, Lloyd Instruments). Li et al., used this method previously to study the effects of ledtrozole-induced changes on bone mineral properties and mechanical functions of laying hens (35).

### Section and HE Staining

The fixed femoral head cartilage was washed thoroughly with PBS and decalcified in 10% EDTA for 1 week at room temperature. After dehydration, the cartilage was embedded in paraffin and cut into 4-μm-thick sections. The sections were stained with hematoxylin and eosin (**HE**) for pathological analysis.

### Biochemical Analysis and ELISA

Frozen plasma was thawed in an ice bath. The levels of alkaline phosphatase (**ALP**), cholesterol (**CHOL**), high-density lipoprotein (**HDL**), low-density lipoprotein (**LDL**), triglyceride (**TG**), calcium (**Ca**), and phosphorus (**P**) were detected with a HITACHI automatic biochemical analyzer (Tokyo, Japan).

The indicators were measured according to the instructions of the ELISA kit (Angle Gene Bioengineering Co., Ltd., Nanjing, China), including bone-specific alkaline phosphatase (**BALP**), carboxy-terminal telopeptide of type-I collagen (**CTX-1**), osteocalcin (**OC**), and tartrate-resistant acid phosphatase 5b (**TRACP5b**).

### Real-Time Quantitative PCR

The femoral head cartilage was grinded into powder in liquid nitrogen (−196°C) and treated with Trizol (Angle Gene Bioengineering Co., Ltd., Nanjing, China) to extract the total RNA. cDNA was synthesized by reverse transcription utilizing HiScript II QRT SuperMix for qPCR (+gDNA wiper; Zazyme, Nanjing, China).

Expression levels of the autophagy-related genes (beclin1, LC3, HIF1α, HIF2α, PI3K, mTOR, AKT, and ATG5) and the specific marker genes (collagen-2, aggrecan) of chondrocytes were detected by quantitative real-time PCR (**qRT-PCR**) on an ABI PRISM 7,300 HT sequence detection system (Applied Biosystems, Inc., Foster City, CA, USA), each sample was repeated 3 times. Quantitative data were normalized relative to the housekeeping glyceraldehyde 3-phosphate dehydrogenase (**GAPDH**) gene. The genes' primer sequences as described above are listed in Table 2. The results were analyzed as the relative fold-change (2^−ΔΔCT^ value) (36).

**Table 2:** Primer sequences in qRT-PCR used to amplify relative mRNAs.

**Target gene**	**Primer sequence (5′−3′)**	
GAPDH	Forward:	GAACATCATCCCAGCGTCCA
	Reverse:	CGGCAGGTCAGGTCAACAAC
Collagen-2	Forward:	ACCTACAGCGTCTTGGAGGA
	Reverse:	ATATCCACGCCAAACTCCTG
Aggrecan	Forward:	TGCAAGGCAAAGTCTTCTACG
	Reverse:	GGCAGGGTTCAGGTAAACG
Beclin1	Forward:	ACCGCAAGATTGTGGCTGAAGAC
	Reverse:	TGAGCATAACGCATCTGGTTCTCC
LC3	Forward:	CCTGGTGCCAGATCACGTCAAC
	Reverse:	AAGCCGTCCTCGTCCTTCTCG
HIF1α	Forward:	CAGCCAGGTGCCGAAGAAGC
	Reverse:	ATGGTCAGCCTCATAATGGATGCC
HIF2α	Forward:	CTGTTGACGATGAGCAGTGCCT
	Reverse:	CCAGGTGTTGGAGCCAGTTGTG
PI3K	Forward:	TCTTCGGATGTTGCCTTACGGTTG
	Reverse:	TTCTTGTCCTTGAGCCACTGATGC
mTOR	Forward:	AACCACTGCTCGCCACAATGC
	Reverse:	GATCGCCACACGGATTAGCTCTTC
AKT	Forward:	GGCTACAAGGAACGACCGCAAG
	Reverse:	TACTGTGGTCCACTGGAGGCATC
ATG5	Forward:	GGCACCGACCGATTTAGT
	Reverse:	GCTGATGGGTTTGCTTTT

*GAPDH, glyceraldehyde 3-phosphate dehydrogenase; LC3, microtubule-associated protein 1 light chain 3; HIF, hypoxia-inducible factor; PI3K, phosphoinositide 3-kinase; mTOR, mammalian target of rapamycin; AKT, protein kinase B; ATG, autophagy-related gene*.

### Statistical Analysis

All data were analyzed using the variance procedure in SPSS 25 (SPSS, Inc., Chicago, IL, USA) and presented as the means ± standard error (SE). The differences among groups were determined by one-way ANOVA (Dunnett's T3). Differences were considered significant at *P* < 0.05.

## Results

### Morbidity of FHN

The evaluations of the walking ability of broilers are shown in Table 3. MP treatment significantly increased the number of broilers which presented lameness (Gait score 3 and 4). Co-treatment with 3-MA reduced the number of broilers with severe gait defect (Gait score 4).

**Table 3:** The evaluation of the walking ability of broilers.

**Gait scoring** **(0–5)**	**Control** **(*n* = 16)**	**3-MA** **(*n* = 16)**	**MP** **(*n* = 16)**	**MP+3-MA** **(*n* = 16)**
0	4	4	0	1
1	7	8	2	6
2	4	4	6	5
3	1	0	4	3
4	0	0	4	1
5	0	0	0	0

*Gait score 0, The bird walked normally with no detectable abnormality; Gait score 1, The bird had a slight defect which was difficult to define precisely; Gait score 2, The bird had a definite and identifiable defect in its gait; Gait score 3, The bird had an obvious gait defect which affected its ability to move about; Gait score 4, The bird had a severe gait defect; Gait score 5, The bird was incapable of sustained walking on its feet (37)*.

The results of FHN evaluation are listed in Table 4. MP treatment increased the incidence of FHN substantially, while co-treatment with 3-MA reduced the occurrence of FHN.

**Table 4:** FHN evaluation and morbidity in broilers, both legs of each broiler were counted.

**Item**		**Control** **(*n* = 32)**	**3-MA** **(*n* = 32)**	**MP** **(*n* = 32)**	**MP+3-MA** **(*n* = 32)**
FHN evaluation score (0–2)	0	29	30	14	27
	1	3	2	11	3
	2	0	0	7	2
Morbidity Score 1–2 (%)		9.38	6.25	56.25	15.63

*FHN evaluation score 0, The normal femoral head with integral structure; FHN evaluation score 1, The femoral head separating from articular cartilage without any visible damage to the growth plate; FHN evaluation score 2, Femoral head separation with laceration of the growth plate (38)*.

### Bone Parameters and Pathological Changes

The bone parameters of the humerus, femur, and tibia are shown in Table 5. There were no significant differences between the 3-MA group and control group (*P* > 0.05). However, the bone density, bone strength, bone length, and bone index of all three types of bones in the MP group were significantly lower than those in the control group (*P* < 0.05). Following co-treatment with 3-MA, the reduction in bone density, bone strength, bone length, and bone index was mitigated, particularly that of bone strength.

**Table 5:** Bone parameters of humerus, femur, and tibia.

	**Parameters**	**Control**	**3-MA**	**MP**	**MP+3-MA**
Humerus	BMD (mg/cm^2^)	226.01^a^±4.44	214.70^a^±8.32	181.17^b^±5.32	186.05^b^±4.90
	Bone strength (N)	189.66^a^±8.73	173.06^a^±14.04	125.99^b^±11.55	169.18^a^±4.24
	Bone length (mm)	68.67^a^±0.71	68.33^ab^±0.76	62.22^c^±0.80	65.30^b^±1.09
	Bone index (g/kg)	2.84^a^±0.07	2.79^a^±0.14	2.33^b^±0.08	2.53^ab^±0.04
Femur	BMD (mg/cm^2^)	186.84^a^±7.10	186.21^a^±5.06	167.98^b^±4.32	180.34^ab^±3.51
	Bone strength (N)	162.72^a^±14.67	140.36^a^±12.26	116.12^b^±5.49	152.43^a^±7.35
	Bone length (mm)	82.45^a^±0.84	81.20^a^±0.79	74.07^b^±0.77	75.60^b^±0.92
	Bone index (g/kg)	5.39^a^±0.13	5.34^a^±0.13	5.07^b^±0.10	5.13^a^±0.09
Tibia	BMD (mg/cm^2^)	219.48^a^±6.65	213.93^ab^±4.07	190.12^c^±6.12	194.48^bc^±5.50
	Bone strength (N)	150.49^a^±7.56	146.36^a^±10.56	120.98^b^±5.83	144.60^a^±4.17
	Bone length (mm)	110.00^a^±1.53	108.70^a^±0.97	100.16^b^±1.03	101.75^b^±1.44
	Bone index (g/kg)	7.84^a^±0.22	7.57^a^±0.27	7.28^b^±0.11	7.49^a^±0.21

*Levels of BMD, bone strength, bone length, and bone index for each group (n = 8/group). All data are shown as the mean ± SE*.

a−c *Indicated superscripts within each group are significantly (P < 0.05) different. 3-MA, 3-methyladenine; MP, methylprednisolone; BMD, bone mineral density*.

**Figure 1:**
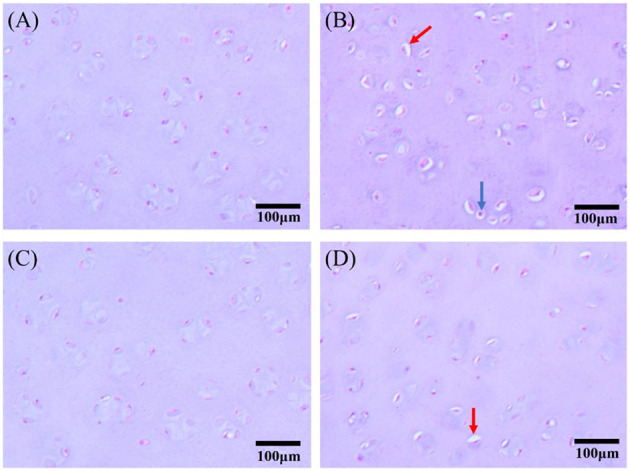
Hematoxylin and eosin (HE) staining of chondrocytes in the femoral head articular cartilage (scale bar: 100 μm). **(A)** Control group: Chondrocytes showed typical features in normal broilers. **(B)** MP group: There was obvious vacuole in the cytoplasm of chondrocyte, the nucleus of which was twisted and squeezed out to the cell edge (red arrow), and karyopyknosis could be observed in some of the chondrocytes (blue arrow). **(C)** 3-MA group: No obvious pathological changes. **(D)** 3-MA+MP group: Vacuoles were observed in fewer chondrocytes compared with MP group (red arrow).

In addition, some femoral heads were selected to prepare paraffin sections and for HE staining (Figure 1). The chondrocytes of broilers in the control group and 3-MA group showed a normal morphology and intact structure. In MP group, obvious vacuole was observed in the cytoplasm of chondrocyte, and the nucleus was squeezed out to the cell edge. When co-treated with 3-MA, this pathological change was alleviated.

### Weights and Blood Biochemistry

**Figure 2:**
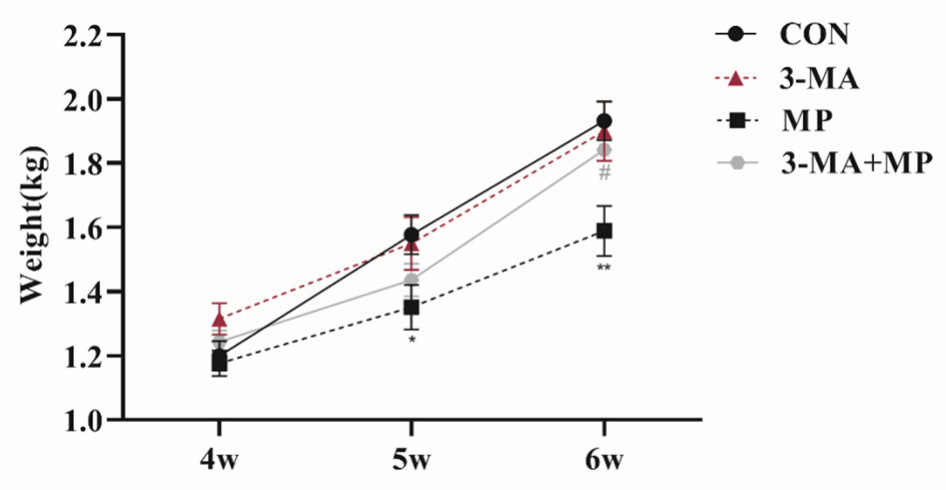
Body weight at 3 aging stages (4, 5, 6 wk) for each group. Treatment with 3-MA caused no significant difference in body weight (*P* > 0.05). Broilers were co-treated with MP and 3-MA. *P < 0.05 vs. control group; **P < 0.01 vs. control group; ^#^*P* < 0.05 vs. MP group. 3-MA, 3-methyladenine; MP, methylprednisolone.

Changes in the body weight of broilers at three different stages (4, 5, and 6 wk) are shown in Figure 2. 3-MA had no evident effect on broiler weight in all stages compared to the control (*P* > 0.05), whereas the body weight of 5 wk (*P* < 0.05) and 6 wk (*P* < 0.01) MP-treated broilers were decreased significantly compared with the control group. Furthermore, after co-treatment with 3-MA, the average weight of broilers was significantly elevated (*P* < 0.05) compared to after MP treatment.

Table 6 shows that 3-MA treatment did not significantly affect the plasma levels of ALP, CHOL, HDL, LDL, TG, Ca, and P (*P* > 0.05). MP treatment significantly decreased the levels of ALP, HDL, Ca, and P and significantly increased the levels of CHOL, LDL, and TG. Moreover, co-treatment with 3-MA effectively mitigated the impacts of MP (*P* < 0.05).

**Table 6:** Laboratory biochemical indicators.

**Parameters**	**Control**	**3-MA**	**MP**	**MP+3-MA**
ALP (10^3^ U/L)	3.93^a^±0.30	3.81^a^±0.46	2.72^b^±0.21	3.92^a^±0.36
CHOL (mmol/L)	2.60^b^±0.05	2.62^b^±0.20	3.07^a^±0.07	2.64^b^±0.03
HDL (mmol/L)	2.22^a^±0.08	2.15^ab^±0.12	1.93^b^±0.05	2.17^a^±0.09
LDL (mmol/L)	0.34^b^±0.02	0.34^b^±0.03	0.47^a^±0.03	0.34^b^±0.02
TG (mmol/L)	0.39^b^±0.01	0.38^b^±0.03	0.46^a^±0.02	0.39^b^±0.01
Ca (mmol/L)	2.56^a^±0.02	2.54^a^±0.04	2.42^b^±0.01	2.53^a^±0.03
P (mg/L)	19.60^a^±0.56	19.27^ab^±0.68	17.66^b^±0.40	19.58^a^±0.22

*Levels of plasma ALP, CHOL, HDL, LDL, TG, Ca, and P for each group (n = 8/group). All data are shown as the mean ± SE*.

a−b *Indicated superscripts within each group are significantly (P <0.05) different. 3-MA, 3-methyladenine; MP, methylprednisolone; ALP, alkaline phosphatase; CHOL, cholesterol; HDL, high-density lipoprotein; LDL, low-density lipoprotein; TG, triglyceride; Ca, calcium; P, phosphorus*.

Table 7 shows that 3-MA treatment significantly decreased the level of OC (*P* < 0.05) and increased the level of CTX-1 (*P* < 0.05), but had no effect on the activities of BALP and TRACP5b compared with the control group (P > 0.05). The activities of BALP and OC in the MP group were significantly lower than those in the control group. After co-treatment with 3-MA, the activities of BALP and OC were clearly elevated (*P* < 0.05). The activities of CTX-1 and TRACP5b in the MP group were significantly higher than those in the control group, and co-treatment with 3-MA significantly reduced the activities of CTX-1 and TRACP5b (*P* < 0.05).

**Table 7:** Levels of plasma bone metabolism parameters.

**Parameters**	**Control**	**3-MA**	**MP**	**MP+3-MA**
BALP (ng/L)	440.29^ab^±15.56	413.18^bc^±2.76	397.08^c^±11.51	455.25^a^±8.72
OC (ug/L)	25.99^a^±1.05	20.58^b^±0.19	17.04^c^±0.94	20.14^b^±0.13
CTX-1 (μg/L)	280.53^b^±13.25	369.51^a^±8.27	388.50^a^±16.00	314.75^b^±11.90
TRACP5b (μg/L)	1.39^bc^±0.03	1.36^c^±0.05	1.57^a^±0.03	1.47^b^±0.02

*Levels of plasma BALP, OC, CTX, and TRACP5b for each group (n = 8/group). All data are shown as the mean ± SE*.

a−c *Indicated superscripts within each group are significantly (P <0.05) different. 3-MA, 3-methyladenine; MP, methylprednisolone; BALP, bone alkaline phosphatase; CTX, carboxy-terminal telopeptide of type-I collagen; OC, osteocalcin; TRACP5b, tartrate-resistant acid phosphatase 5b*.

### Expression of Relative Factors on Autophagy

**Figure 3:**
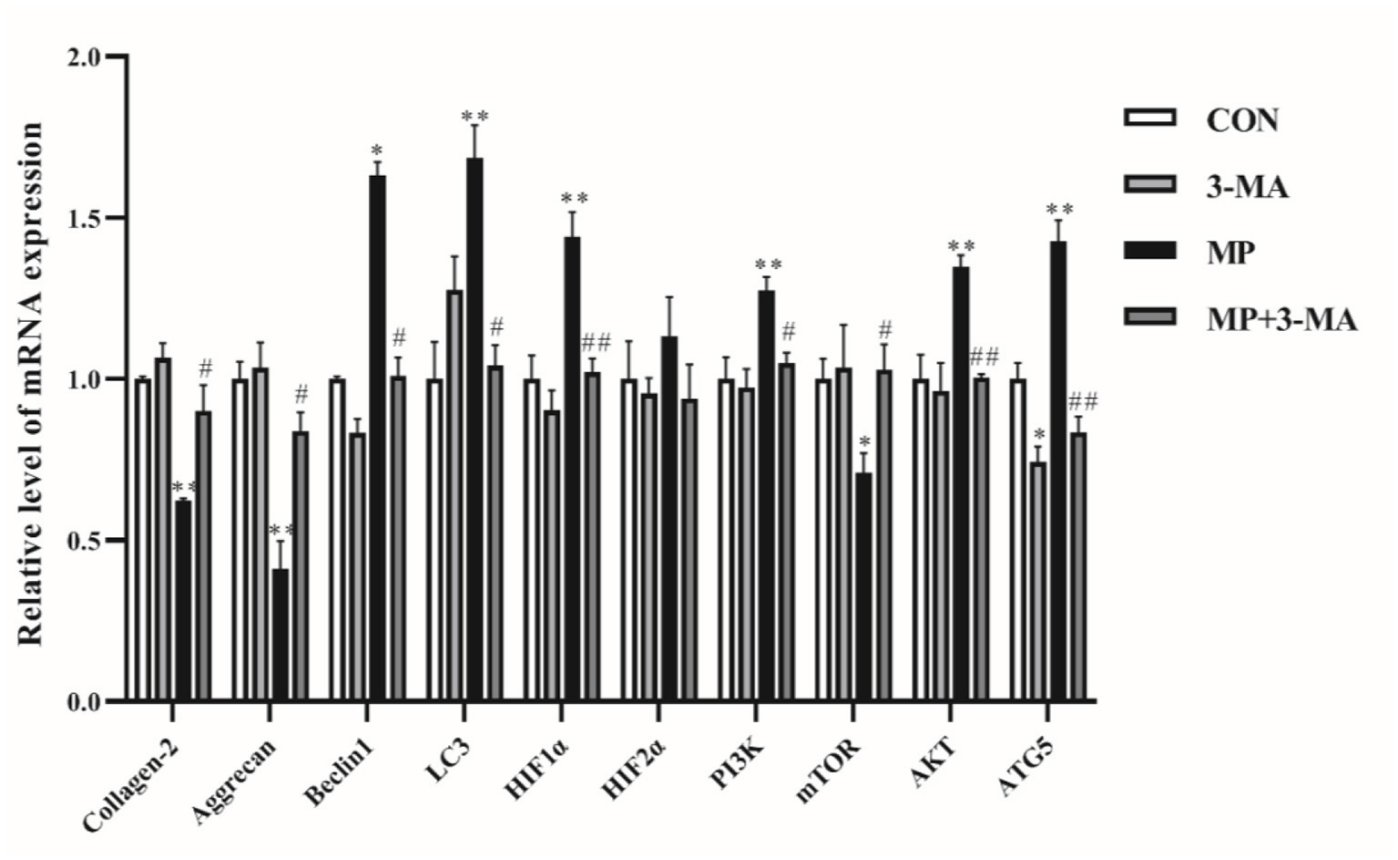
mRNA expression of autophagy relative genes. Effects of 3-MA alone were examined by reverse transcription-quantitative polymerase chain reaction. No significant difference was detected (*P* > 0.05). Broilers were co-treated with MP and 3-MA. *P < 0.05 vs. control group; **P < 0.01 vs. control group; ^#^*P* < 0.05 vs. MP group; ^##^*P* < 0.01 vs. MP group. 3-MA, 3-methyladenine; MP, methylprednisolone.

qRT-PCR was performed to examine the mRNA expression differences of target genes (Figure 3). The expression of collagen-2 and aggrecan reflected the ability of chondrocytes to secrete extracellular matrix. Beclin1 and LC3 genes played a crucial role in autophagy. Compared with the control group, the mRNA expression of collagen-2, aggrecan, and LC3 was increased, whereas that of beclin1 was decreased following treatment with 3-MA alone (*P* > 0.05). After exposure to MP, the expression of collagen-2, aggrecan and mTOR was significantly lower than that in the control group. In addition, the expression of beclin1, LC3, PI3K, AKT, and ATG5 was significantly increased compared with the control group. Furthermore, co-treatment with 3-MA and MP significantly upregulated the expression of collogen-2, aggrecan and mTOR and decreased the expression of beclin1, LC3, HIF1α, PI3K, AKT, and ATG5 compared to these levels in the MP-treated group. These results indicated that beclin1-dependent autophagy was overactivated and occurred after MP treatment.

## Discussion

FHN in broilers involves multiple factors such as genetic susceptibility, diet, age, and environment (39). Particularly, the growth rate of bones cannot keep up with the body weight gains in broilers and may result in a greater inherited tendency for skeletal problems (40, 41). Based on these characteristics, broilers are suitable for use as experimental models of FHN induced by MP (13, 42).

Bone quantity after GC treatment can be evaluated by measuring bone parameters (43, 44). The significant reduction in BMD, bone strength, bone length, and bone index indicated that MP inhibited the growth of bones and caused bone mineral loss (45). Previous studies in poultry also demonstrated that MP caused bone loss and reduced bone quality (30, 46). We found that 3-MA treatment alleviated the negative effects of MP treatment on bone quality in broilers.

The levels of various chemicals in the blood are classically used as indicators of abnormal states, with biochemical bone markers sensitively reflecting bone molding and remolding, and are useful for studying the pathophysiology of bone metabolism in response to GC administration (47). Under MP treatment, bone formation indicators (BALP and OC) were lower than in the control group, whereas bone resorption indicators (CTX-1 and TRACP5b) were higher (37, 48). These observations were consistent with those of previous studies (38, 49), in which GC treatment was shown to impair bone formation and enhance bone resorption (50). We found that 3-MA mitigated the effects of MP treatment on blood chemistry in broilers and that autophagy regulated bone formation and bone resorption.

Aggrecan and collagen-2 are specific markers of chondrocytes and cartilage (51, 52). We found that the mRNA expression of collagen-2 and aggrecan was significantly decreased in MP group, which was consistent with previous study in broilers, the secretory activity of chondrocytes may be seriously impaired (13).

3-MA treatment may inhibit PI3K/AKT signal pathway by inhibiting PI3K and intervene the expression of autophagy gene in articular chondrocytes induced by MP. MP may have overactivated chondrocyte autophagy, as we observed that MP treatment significantly increased the expression of both beclin1 and LC3 of the femoral head (53). Treatment with the autophagic inhibitor 3-MA significantly suppressed the expression of autophagy-related genes, which induced by MP in broilers. However, autophagy is considered as a double-edged sword, and the effect of autophagy in GC-treated osteonecrosis remain controversial. During the initial autophagy process, cells may remain viable during periods of metabolic stress. However, higher and persistent stress may generate high accumulation of autophagosomes, leading to cell death. Previous studies showed that dexamethasone induced apoptosis in chondrocytes, and autophagy protected chondrocytes from GC-induced apoptosis via ROS/AKT/FOXO3 signaling (54). Additionally, activation of osteocyte autophagy was found to be significantly increased when the cells were treated with a low dose of GC; higher doses of GC activated the gene pathway for osteocyte apoptosis (15).

## Conclusion

In summary, overactivated autophagy occurs after MP treatment in broilers, greatly reducing their bone quality which is primarily manifested as reduced BMD and bone strength. Induction of high levels of autophagy by MP may occur *via* the beclin1-dependent autophagy signal pathway. Furthermore, this study provides new directions for the treatment and prevention of femoral head necrosis induced by glucocorticoids.

## Data Availability Statement

The original contributions presented in the study are included in the article/supplementary material, further inquiries can be directed to the corresponding author/s.

## Ethics Statement

The animal study was reviewed and approved by Institutional Animal Care and Use Committee of Nanjing Agricultural University.

## Author Contributions

KP: performed the experiments. SW: arranged the data for statistical analysis. ML: drafted and revised the manuscript. ZZ: designed the experiments and revised the manuscript. All authors have read and approved the final manuscript.

## Funding

This work was supported by the National Natural Science Foundation of China (Grant Number 32072936) and a project funded by the Priority Academic Program Development of Jiangsu Higher Education Institutions (PAPD).

## Conflict of Interest

The authors declare that the research was conducted in the absence of any commercial or financial relationships that could be construed as a potential conflict of interest. The reviewer KL declared a shared affiliation, though no other collaboration, with the authors KP, SW, ML, and ZZ, to the handling editor.

## Publisher's Note

All claims expressed in this article are solely those of the authors and do not necessarily represent those of their affiliated organizations, or those of the publisher, the editors and the reviewers. Any product that may be evaluated in this article, or claim that may be made by its manufacturer, is not guaranteed or endorsed by the publisher.

## Abbreviations

MP: methylprednisolone
3-MA: 3-methyladenine
GCs: glucocorticoids
FHN: femoral head necrosis
ERs: endoplasmic reticulum stress
LC3: light chain 3
HIF: hypoxia-inducible factor
PI3K: phosphoinositide 3-kinase
mTOR: mammalian target of rapamycin
AKT: protein kinase B
ATG: autophagy-related gene
BMD: bone mineral density
HE: hematoxylin and eosin
ALP: alkaline phosphatase
CHOL: cholesterol
HDL: high-density lipoprotein
LDL: low-density lipoprotein
TG: triglyceride
Ca: calcium
P: phosphorus
BALP: bone-specific alkaline phosphatase
CTX-I: carboxy-terminal telopeptide of type-I collagen
OC: osteocalcin
TRACP5b: tartrate-resistant acid phosphatase 5b
qRT-PCR: quantitative real-time PCR
GAPDH: glyceraldehyde 3-phosphate dehydrogenase.
